# Antidiabetic and Neuroprotective Effect of the N-Butanol Extract of* Fragaria nilgerrensis* Schlecht. in STZ-Induced Diabetic Mice

**DOI:** 10.1155/2018/6938370

**Published:** 2018-09-04

**Authors:** Liangcai Gao, Xinyi Wang, Zejie Lin, Ningning Song, Xinnan Liu, Xinxin Chi, Tiange Shao

**Affiliations:** School of Life Science, East China Normal University, Shanghai 200241, China

## Abstract

Diabetes has been associated with neurodegenerative disorders that are accompanied by memory loss and cognitive impairments, but there is no effective treatment for it at present.* Fragaria nilgerrensis *Schlecht. (FNS), a well-known Chinese materia medica, has been traditionally used for the folkloric treatment of diabetes and other diseases. However, its effects are poorly documented. Here, we investigated the antidiabetic and neuroprotective effect of FNS in diabetic mice. Thin layer chromatography (TLC) and high performance liquid chromatography (HPLC) evaluations of N-butanol extract of* Fragaria nilgerrensis *Schlecht. (N-FNS) showed the presence of flavonoid and its structure is similar to scutellarin. For the first time, we show the potential neuroprotective and antidiabetic effects of FNS. After 4 weeks of FNS intervention, a significant decrease in blood glucose, increase in body weight, and amelioration in glucose tolerance were observed in FNS treated diabetic mice. In the acute study, FNS enhanced motor activity in the open field task and significantly prevented spatial-learning deficits in Morris water maze tests. Besides, synapse ultrastructure of the hippocampus showed that the mitochondrial morphology was basically restored and all the synaptic structural parameters were gradually normalized after treatment with FNS. Importantly, we found that the activities of SOD and CAT in liver and hippocampus of diabetic mice significantly increased after FNS administration. In vitro, FNS and scutellarin showed high DPPH radical scavenging activity. The study suggests that FNS exerted significant antidiabetic and neuroprotective effects which may be attributed to its antioxidant property.

## 1. Introduction

Diabetes mellitus is a chronic metabolic disease characterized by hyperglycemia which can lead to a series of complications [1, 2], including pancreatic diseases, cardiovascular disease, liver diseases, blindness, and neurodegenerative diseases [3]. Links between neurodegenerative diseases such as Alzheimer's disease and diabetes have been well established. The deposition of advanced glycation end products does not spare the brain and they have been found in senile plaques, where they can reduce the solubility of proteins such as beta amyloid and Tau proteins. Some authors favour the hypothesis of brain insulin resistance because, in a few small studies, insulin was found to improve memory [4, 5]. Compared with those without diabetes, those with the diseases have a 1.2- to 1.5-fold greater rate of decline in cognitive function [6]. A number of clinical and epidemiological studies have shown that the pathophysiological features of diabetes and neuropathic disease are similar to each other, which share complex and linked mechanism including insulin resistance, inflammation, and oxidative stress [7–9]. Besides, the impairment of insulin signaling in the brain may damage the ability of neurons to self-repair and could enhance the development of neurodegenerative disorders [4, 10].

However, scientific evidence to support an appropriate treatment for diabetes with cognitive impairment is scarce. Therefore, to investigate a drug which has both antidiabetic effect and cognitive protective ability is a crucial issue.

Plants play a major role in discovering new therapeutic agents [11] and have been considered as sources of biologically active substances including antioxidants and hypoglycemic and neuroprotective agents [12, 13].* Fragaria nilgerrensis *Schlecht. (FNS), commonly known as perennial herbaceous, is a member of the Rosaceae family and is widely cultivated in China, Nepal, Sikkim, Eastern India, and Northern Vietnam. Folk medicine and *⟪*medicinal plant dictionary*⟫* have revealed that FNS has high medical properties. Plant species belonging to family Rosaceae have been demonstrated to be effective against cancer, diabetes, and other diseases. However, the pharmaceutical effects of FNS are scarcely explored. In this study, we investigated the antidiabetic ability of FNS, assessed the radical scavenging activity of FNS and its major constituent scutellarin, evaluated its protective effect in learning and memory formation, and observed the influence on the microstructure change of the hippocampus in STZ-induced mice.

## 2. Materials and Methods

### 2.1. Chemicals

Streptozotocin (STZ) was obtained from Sigma Chemicals (St. Louis, USA); superoxide dismutase (SOD), catalase (CAT), and malondialdehyde (MDA) test kits were purchased from Nanjing Jiancheng Bioengineering Institute (Nanjing, China). All the other chemicals used in the study were obtained from Sinopharm Chemical Reagent Beijing Co, Ltd (Beijing, China).

### 2.2. N-Butanol Extracts of FNS

The* Fragaria nilgerrensis *Schlecht. was collected in February 2016, Yunnan, and authenticated by the Herbarium Department of the College of Life Science, East China Normal University. For preparation of the extract, the root, stem, and leaves of* Fragaria nilgerrensis *Schlecht. were dried and crushed into powder. Briefly, each 1 g of powder was soaked in 75% of 70 ml N-butanol at room temperature for 48 h. Then, the filtrates were concentrated under reduced pressure using a rotary evaporator at 40°C until extraction solvent was completely dried. The final extracts were stored at 4°C for use in subsequent experiments. The yield of the N-butanol extract was 3.323% (w/w)

### 2.3. Animals

Adult Kunming male mice (body weight: 20±2g) were purchased from the Experimental Animal Center of East China Normal University (Certificate: AR2013108002).The colony was housed in an ambient temperature of 23±3°C and maintained on a 12 h light/dark cycle with normal laboratory chow standard pellet diet [14]. All the studies were approved by the Institutional Animal Care and Use Committee of East China Normal University and in accordance with the criteria outlined in the Guide for the Care and Use of Laboratory Animals published by the National Institutes of Health.

### 2.4. Preliminary Phytochemical Screening of N-FNS

The preliminary phytochemical analysis was carried out for N-FNS, using standard phytochemical methods.

### 2.5. Total Flavonoid Content

Total flavonoid was estimated according to the method as follows: 500 ml of N-butanol extract was added to 1 ml of 5% NaNO_3_ into microcentrifuge tube. After 6 min, 1 ml of 10% AlCl_3_ was added followed by 10 ml of 1 M NaOH solution, and the total volume was made up to 25 mL with ethanol. The absorbance was read at 335 nm after 15 min. Scutellarin was used as reference compound. The chemical constituents of FNS were chromatographed on a silica gel.

### 2.6. Flavonoids Characterization by Thin Layer Chromatography (TLC)

2 mg/ml concentration of each sample (N-butanol extract, scutellarin, Nobiletin and Quercetin) was made in methanol and 10 *μ*l loaded onto the TLC plate. The solvent system was standardized as methanol: trichloromethane: acetic acid: water (4:5:1:1, v/v/v/v). The plate was dried and the compounds were observed under UV light.

### 2.7. HPLC Analysis of FNS

The high performance liquid chromatography (HPLC) analysis of the FNS was carried out by the method of Sunil et al. [15]. The N-butanol extract of FNS was analyzed on an analytical Eclipse XDB-C18 using a Luna C18 column (4.6 × 250mm, 5mm). The mobile phase used was 0.06% methanol and phosphoric acid solution (where methanol  :  phosphoric acid = 1  :  1). Flow rate and injection volume of sample were 1.0ml/min and 10 ul, respectively. The results were obtained with UV detection at 360 nm at ambient temperature.

### 2.8. Experimental Design

Ten mice were randomly separated as the normal control group (NC) after acclimatization to the environment for 1 week, and diabetes was induced by a single intraperitoneal injection of streptozotocin (STZ) at 40 mg/kg in 0.4% citrate buffer. On the seventh day after STZ injection, mice with fasting glycaemia between 15 mmol·L^−1^ and 30 mmol·L^−1^ were verified as diabetic mice [16]. Then, the diabetic mice were randomly divided into four groups of ten mice each as follows: diabetes mellitus group (DM), metformin group (Metformin), low-dose FNS group (L-FNS), and high-dose FNS group (H-FNS). Each group was treated at 10 ml/kg by means of intragastric administration, which was processed with a syringe daily. DM and NC group were treated with pure water. Metformin group received 150 mg/kg b.w, which was used as the positive control. L-FNS and H-FNS group were, respectively, treated with 400 mg/kg and 800 mg/kg b.w N-FNS. All mice were provided with sufficient water and food every day until the end of the experiment. At the end of the experiment, the animals were fasted for 12 h, anaesthetized using diethylether, and sacrificed by decapitation.

### 2.9. Acute Toxicity Study

Acute toxicity study was performed according to the previous report [17]. The animals were fasted overnight and provided only with water. Then they were randomly divided into five groups of six animals each which were treated with increased doses of 2000 mg/kg, 3000 mg/kg, 4000 mg/kg, and 5000 mg/kg. One group was considered as control group and was given vehicle alone. After 24 hours, respiratory distress, emaciation, posture, and mortality of all animals were recorded.

### 2.10. Measurement of Body Weight, Oral Glucose Tolerance Test, and Blood Glucose Level

Body weight and fasting blood glucose were measured weekly [18, 19]. The mice were fasted 6 h and the blood sample was withdrawn from the tail vein for fasting blood glucose test. The mice were fasted over 5 h at the end of FNS treatment. A single dose of glucose (2 g/kg b.w) was orally administered to mice, and glucose was measured at 0, 30, 60, 90, and 120 min after glucose treatment. The area under the curve (AUC) between 0 and 120 min during OGTT for the total glucose (AUC Glucose 0–120) was calculated by the trapezoidal method.

### 2.11. Behavioral Procedure

#### 2.11.1. Morris Water Maze Apparatus

At the end of drug treatment, all mice performed the Morris water maze test as described in previously study [20, 21]. The water maze consisted of a circular pool with a diameter of 140 cm and a height of 70 cm, which was filled with room-temperature (25±2°C) water to the depth of 20 cm and a circular dark escape platform (10cm in diameter) submerged 2 cm beneath the surface of the water. Several objects or geometric images such as circles, squares, and triangles with different colors were hung on the wall in the testing room as visual spatial cues. All mice were trained for 5 sequential days with the platform in a fixed position. Mice were placed in the pool from 1 of 4 entrance points (N, E, S, or W) and allowed 60 s to locate the hidden platform. Any mice that did not reach the platform within 60 s were allowed to remain on the platform for 10s. After 48 h from the final training trial, the mice were given a 60 s probe trial without the platform in the pool. The time that the animal spent in currently “correct” quadrant was recorded as an index of reference memory. A video tracking system was mounted above the center of the maze in order to record the motion of mice. Latency to find the platform and traveled distance as well as the swimming strategies were automatically recorded by the CCD then analyzed by software.

#### 2.11.2. Open Field Test

Locomotor activity was measured using the open field test as previously described [22, 23]. Briefly, the apparatus is a 75 × 75 × 40 cm square arena that was divided into 25 equal squares (15 × 15 cm) on the floor of the arena. Each mouse was placed in the central square and observed for 5 minutes, testing in the apparatus once. Scores were calculated by the amount of time, including spent rearing (defined as standing upright on its hind legs) and the number of crossings in the grid lines (it crossed at least three paws)

### 2.12. Electron Microscopy

The hippocampus was rapidly removed, dissected, and then fixed in freshly prepared fixative containing 2.5% glutaraldehyde in sodium cacodylate buffer (0.1 M, pH 7.2). After rinsing in sodium cacodylate buffer, the hippocampus was fixed at 4°C for 2 h in 1% OsO4 in 0.2M Na2HPO3 (phosphate buffer) adjusted to pH 7.3 with HCl [24–26]. Fixed samples were later rinsed with two changes of deionized water and dehydrated in graded steps of alcohol. After infiltration, samples were cut in a frontal plane on an ultramicrotome and collected on copper slot grids coated with pioloform. Sections were contrasted with 2% uranyl acetate (10 min) followed by lead citrate. The hippocampus was identified with a light microscope Leica MM AF, cut out from the coronal slices, dehydrated in graded series of ethanol and acetone, and embedded in araldite. Blocks were trimmed and 70–75-nm-thick sections were cut with an ultramicrotome, picked up on 200-mesh copper grids, double-stained with uranyl acetate and lead citrate, and examined with H7700 transmission electron microscopes. Asymmetric spine synapses were counted according to the rules of the dissector technique within an unbiased counting frame superimposed onto each electron micrograph. The average volumetric density (synapse/*μ*m^3^) of spine synapses within each sampling area was then determined by dividing the sum of spine synapses counted in all samples taken from that particular sampling area by the dissector volume. Dissector volume was calculated by multiplying the area of the unbiased counting frame (79 *μ*m^2^) by ultra section thickness (average 75 nm) and by the number of dissectors. Finally, the volumetric density of spine synapses was multiplied by the volume of the sampling area, determined earlier, to arrive at the total estimated number of spine synapses [27].

### 2.13. Biochemical Analysis

At the end of the experiment, livers and hippocampus were rapidly removed and immediately homogenized (1:9, w/v) in phosphate buffer (0.2M, PH 7.4, 4°C). After centrifugation (4,000 rpm, 4°C) for 20 min, the supernatants were collected for further biochemical analysis. The activities of SOD and CAT, as well as MDA content, were determined using commercial reagent kits according to the instructions.

### 2.14. DPPH Radical Scavenging Assay

The radical scavenging activity of the extract of FNS and scutellarin was determined by DPPH radical scavenging assay [28]. *α*, *α*-diphenyl-*β*-picrylhydrazyl (DPPH) was dissolved in ethanol and then mixed with the extract of FNS and scutellarin. The absorbance was measured at 525 nm with a spectrophotometer (Ultrospec 3000, ECNU, China). Mean values were obtained from triplicate of experiment values. Percentage inhibition was calculated by using the following equation: % inhibition = [(A_0_− A)/A_0_] × 100 (where A_0_ is the absorbance of the control and A is the absorbance of the test sample). VC was used as the standard antioxidants, and the same holds in the following section.

### 2.15. Statistical Analysis

GraphPad Prism for windows version 6 was used for all data and statistical analyses. All values are expressed as means ± SD. Statistical analyses were performed by one-way ANOVA, followed by Dunnett's test. Values were considered to be significantly different when the* P* value was less than 0.05.

## 3. Results

### 3.1. Total Flavonoid Content of N-FNS

**Figure 1:**
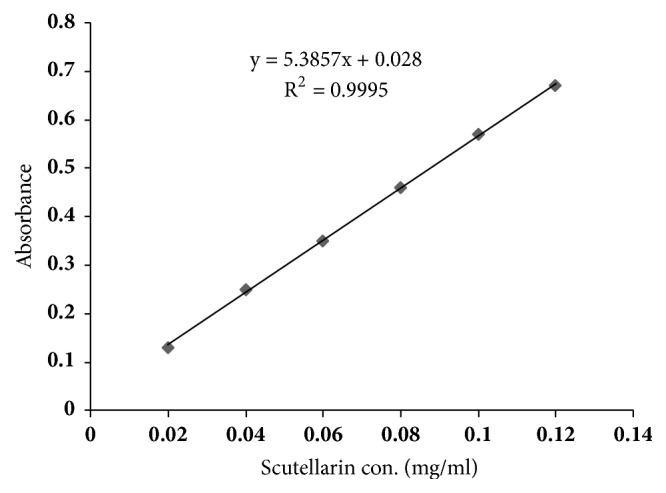
Concentration-response curve of scutellarin standard at 335 nm.

The preliminary phytochemical evaluation of N-FNS showed the presence of flavonoid and triterpene. As shown in Figure 1, the total flavonoid content of N-FNS was 52.31% calculated from scutellarin calibration curve.

### 3.2. TLC Analysis of N-FNS

By TLC, the retention factor (Rf) values of scutellarin, nobiletin, and quercetin spots were, respectively, 0.35, 1.00, and 0.92. The Rf value of N-FNS spot was 0.35, the same as Rf value of scutellarin. To confirm the presence of scutellarin, additional analyzes were performed by HPLC.

### 3.3. HPLC Analysis of N-FNS

**Figure 2:**
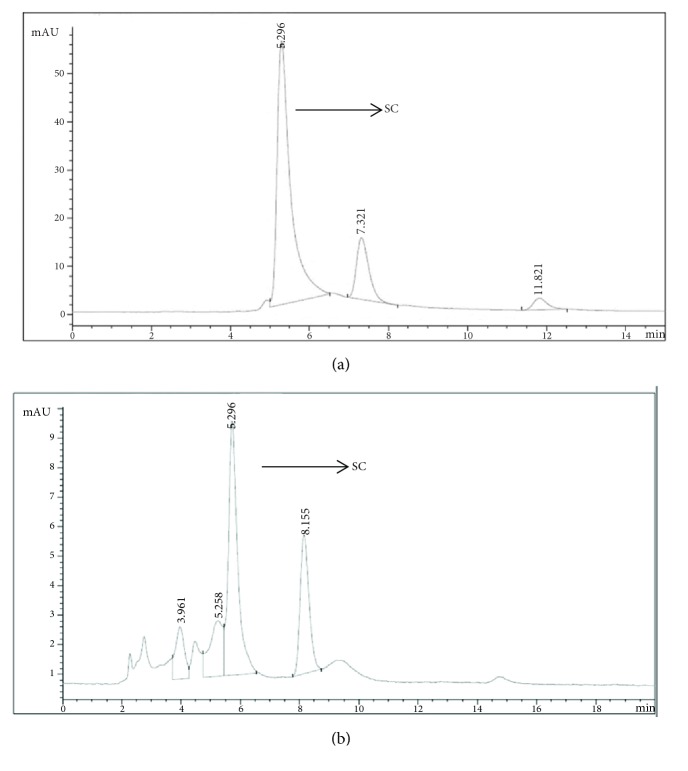
HPLC chromatograms of the standards scutellarin (a) and N-FNS (b) detected at 360 nm. Peak, SC: scutellarin.

The presence of flavonoid in FNS dry extract was established by HPLC. As shown in Figure 2, the baseline was stable under the 360 nm detection. The retention time of scutellarin was 5.296 min (Figure 2(a)). N-FNS had a peak at 5.296 min that is equivalent to scutellarin indicating the same structure as scutellarin (Figure 2(b)), quantified as 64.5%.

### 3.4. Effect of FNS on Body Weight, Blood Glucose Level, and Oral Glucose Tolerance Tests

**Figure 3:**
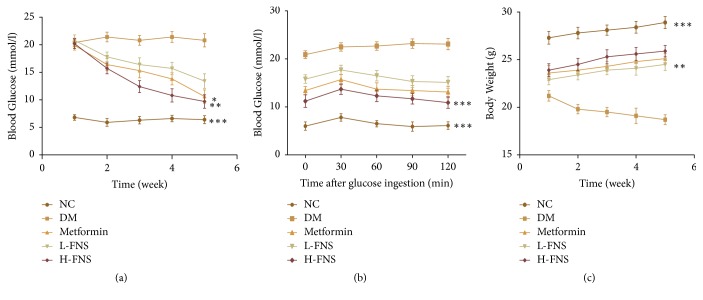
Effect of FNS on (a) blood glucose in diabetic mice, (b) oral glucose tolerance test, and (c) body weight. The results are expressed as mean ± SD (n = 10). ^*∗*^*P<*0.05, ^*∗∗*^*P<*0.01, and ^*∗∗∗*^*P<*0.001 versus DM.

As demonstrated in Figure 3, diabetic mice showed a higher blood glucose (*p*<0.001) (Figure 3(a)), worse glucose tolerance (*p*<0.001) (Figure 3(b)), and rapider body weight loss (*p*<0.001) (Figure 3(c)) when compared with normal control mice. Administration of FNS significantly decreased blood glucose (*p*<0.01) and increased body weight (*p*<0.01) of diabetic mice. After oral administration of 2 g/kg b.w of glucose within 120 min, FNS extractions obviously improved mouse glucose tolerance and significantly decreased blood glucose (*p*<0.001). In brief, the elevation of blood glucose and the impairment of glucose tolerance as well as body weight loss could be prevented with the increase of FNS dosages. The area under the curve (AUC) glucose value in the diabetes mellitus (DM) group (Table 1) was significantly higher than that in normal control (NC) group (*p*<0.001). Meanwhile, the AUCs in metformin group (Metformin), low-dose FNS group (L-FNS), and high-dose FNS group (H-FNS) groups were significantly lower than that in DM group (*p*<0.01).

### 3.5. Effects of FNS on Memory Relearning and Behavior of the Water Maze Task and Open Field Test in STZ-Induced Diabetic Mice

**Figure 4:**
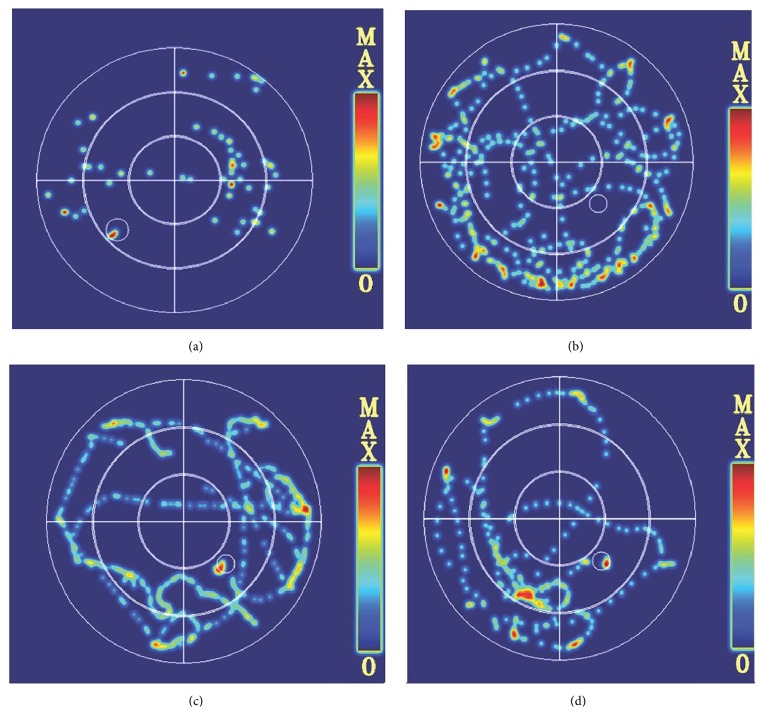
The adaptive swimming trajectory of mice in the Morris water maze. (a) Normal control group. (b) Diabetes mellitus group. (c) Metformin group. (d) H-FNS group.

As shown in Figure 4, no difference in average swimming velocity was observed among all experimental groups, but we found that diabetic mice preferred to swim along the pool perimeter compared with normal control group (Figures 4(a) and 4(b)). However, after treatment with metformin (Figure 4(c)) and FNS (Figure 4(d)), the irregular search pattern of diabetic mice became regular and direct.

**Figure 5:**
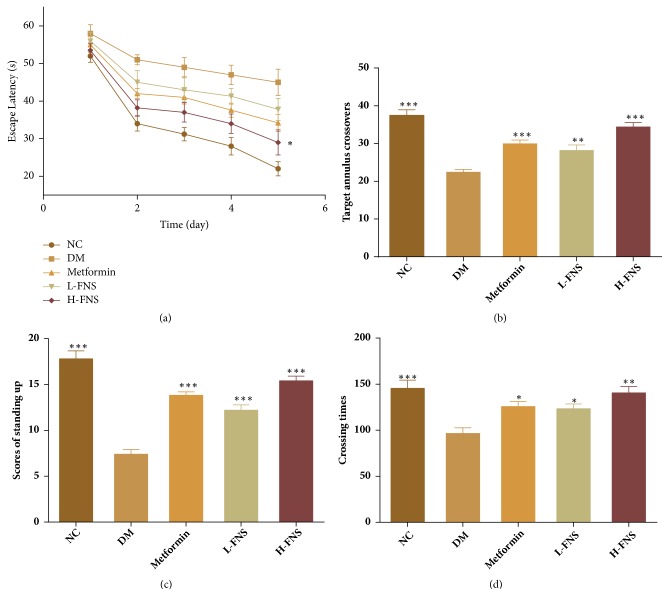
Effects of FNS on memory relearning and behavior of the water maze task and open field test in STZ-induced diabetic mice. (a) Escape latency to find the hidden platform for 5 consecutive days. (b) The number of crossings in target annulus. (c) Number of crossings during the 5 min test in the open field. (d) Scores of standing up during the 5 min test in the open field. The results are expressed as mean ± SD (n = 10). ^*∗*^*P*<0.05, ^*∗∗*^*P*<0.01, and ^*∗∗∗*^*P*<0.001 versus DM.

Over the five days in Morris water maze, as compared with normal control group, the time to find the hidden platform (escape latency) of diabetes mellitus group (DM), as shown in Figure 5(a), was significantly delayed (*p*<0.05). However, FNS group showed a dramatic improvement, and high dosage of FNS showed the most rapid improvement compared with DM (*p*<0.05). Additionally, the results of the probe test also showed prominent memory impairment in STZ-induced diabetic mice. The number of annulus crossings of DM group was significantly decreased compared with normal control group (*p*<0.001). However, this situation was meaningfully reversed by FNS administration, and it would be enhanced at the high-dosage FNS (*p*<0.001) (Figure 5(b)). Besides, the number of crossings in the grid lines (*p*<0.01) and standing up (*p*<0.001) of open field test was significantly increased by FNS and they were also dosage-dependent (Figures 5(c) and 5(d)). The results indicated that the impairment of spatial-learning and passive avoidance performance was attenuated by FNS hypodermic injection.

### 3.6. Effect of FNS on Synaptic Alterations in the Hippocampus of STZ-Induced Diabetic Mice

**Figure 6:**
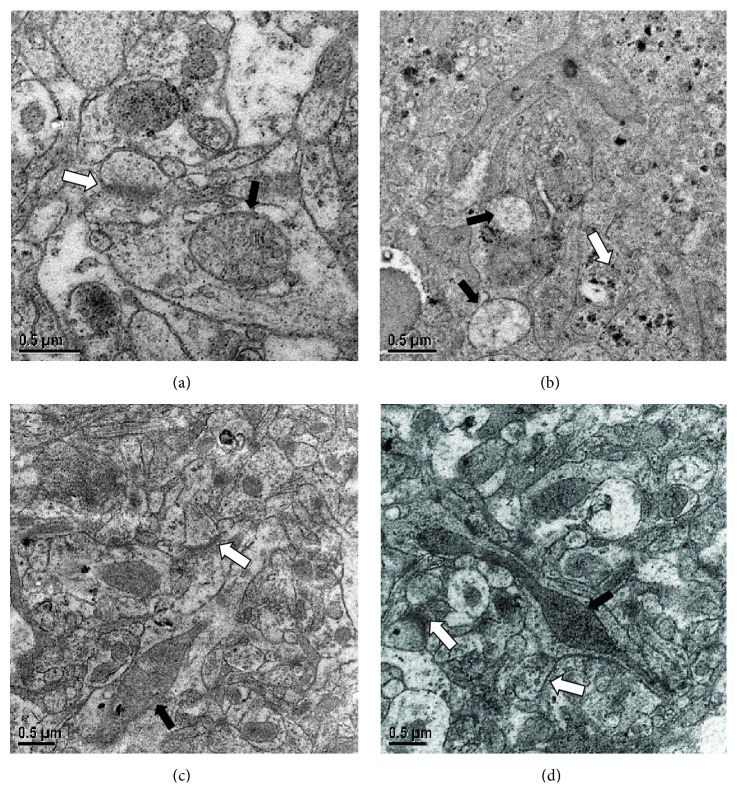
Effect of FNS on synaptic alterations in the hippocampus of STZ-induced diabetic mice. (a) Normal control group. (b) Diabetes mellitus group. (c) Metformin group. (d) H-FNS group. Black arrow marks the mitochondria. White arrow marks the synapsis.

**Figure 7:**
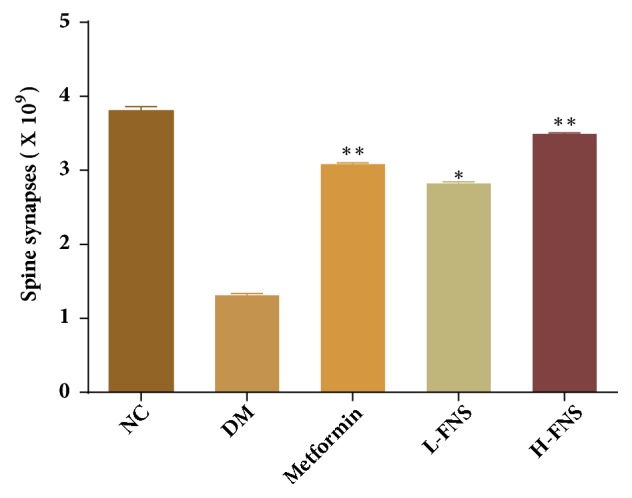
Effect of FNS on the number of spine synapses in hippocampal cell. The results are expressed as mean ± SD (n = 10). ^*∗*^*P*<0.05, ^*∗∗*^*P<*0.01, and ^*∗∗∗*^*P*<0.001 versus DM.

As electron microscopic examination showed, in DM group (Figure 6(b)), the synaptic structure and number in CA1 section of the hippocampus were abnormal, and the mitochondria envelope and cristae appeared to damage. Besides, the front and back membranes of the synapses were unclear when compared to NC group (Figure 6(a)). Additionally, the number of synaptic vesicles in DM group is also greatly reduced. However, the mitochondria morphology was basically restored and all the synaptic structural parameters were gradually normalized in metformin group and H-FNS group (Figures 6(c) and 6(d)). As shown in Figure 7, the number of synapses was significantly increased after treatment with H-FNS (*p*<0.01).

### 3.7. Effect of FNS on the Activities of SOD, CAT, and MDA of Liver and Hippocampus in STZ-Induced Diabetic Mice

**Figure 8:**
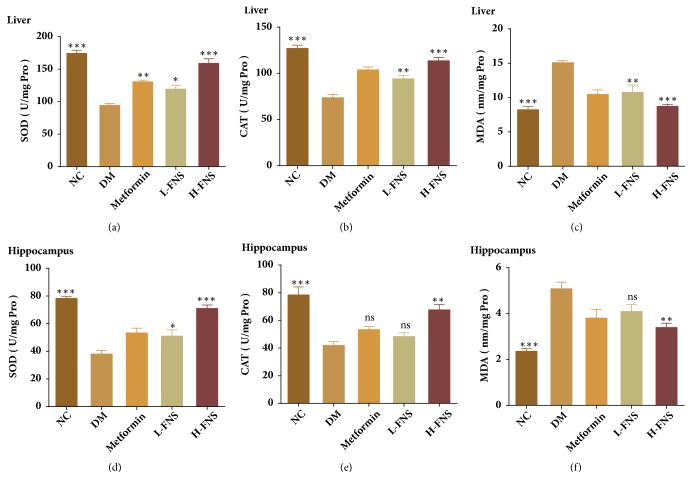
Effect of FNS on the activities of SOD, CAT, and MDA of liver and hippocampus in STZ-induced diabetic mice. The activities of SOD, CAT, and MDA of liver (a, b, c). The activities of SOD, CAT, and MDA of hippocampus (d, e, f). The values are reported as the means ± SD (n = 10). Asterisks denote the significant differences from the diabetes mellitus group at ^*∗*^*P* < 0.05, ^*∗∗*^*P* < 0.01, and ^*∗∗∗*^*P*<0.001 versus DM.

As Figure 6 shows, compared with NC group, the activity of SOD and CAT of DM group was significantly depleted in liver (*p*<0.001, respectively) (Figures 8(a) and 8(b)) and hippocampus (*p*<0.001, respectively) (Figures 8(d) and 8(e)). Meanwhile, obvious increase of malonaldehyde (MDA) in DM group was observed in liver (p<0.001) (Figure 8(c)) and hippocampus (p<0.001) (Figure 8(f)). In particular, after treatment with FNS, MDA level was decreased in liver (p<0.01) and hippocampus (Figures 8(c) and 8(f)) and SOD and CAT activities were increased in liver (p<0.05, respectively) (Figures 8(a) and 8(b)) and hippocampus (Figures 8(d) and 8(e)).

### 3.8. Effect of Scutellarin on DPPH Radical Scavenging Activity

**Figure 9:**
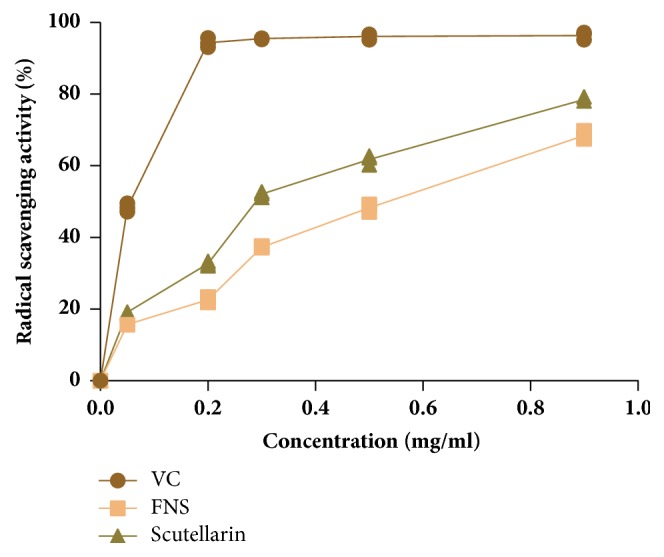
Effect of scutellarin on radical scavenging activities. The results are expressed as mean ± SD (n=3). VC: ascorbic acid.

As seen in Figure 9, both FNS and its constituent scutellarin exhibited obvious DPPH radical scavenging activity. Compared with VC with IC_50_ values of 0.065mg/ml, IC_50_ values of FNS and scutellarin are 0.28 mg/ml and 0.51 mg/ml, respectively. And the antioxidant activities of FNS and scutellarin appeared obviously as the dose-dependent relationships.

## 4. Discussion

In the present study, we analyzed the effect of* Fragaria nilgerrensis *Schlecht. (FNS), a traditional Chinese medicine, on learning and memory performance and microstructure change of the hippocampus as well as the biochemical function of diabetic mice. STZ-induced diabetic mice in this study developed impairment in cognitive function which was associated with a significant decrease in SOD and CAT activity in the hippocampus. Administration of FNS significantly and dose dependently ameliorated cognitive deficits, cholinergic dysfunction, and oxidative stress in diabetic mice.

STZ-induced diabetes is a well-documented model of experimental diabetes which produces hyperglycemia and severe loss in body weight [29, 30]. The decrease in body weight is due to the increased muscle destruction or degradation of structural proteins [31]. In the present study, it showed an obvious rise in serum glucose and a significant decrease in body weight in diabetic mice which are consistent with previous studies. However, we found that treatment with FNS decreased the blood glucose level and also improved body weight loss in diabetic mice. Besides, glucose tolerance is another major characteristic of metabolic syndrome. Treatment with FNS improved glucose regulation in the diabetic mice. These results suggested that diabetes was well-controlled by administration of FNS.

In the present study, the Morris water maze was used for the assessment of learning and memory [20, 21]. STZ-induced diabetes produced marked impairment in cognitive function [32]. We found that diabetic mice showed a poor performance in learning and memory test, as they had longer latency to reach the hidden platform compared to control group. The latency is associated with the time the input signal takes to reach the involved brain structures (synaptic delays) and is measuring the integration and association processes of the signal during the acquisition phase [24, 33]. Furthermore, the present results indicated that the impaired performance of diabetic mice is related to cognitive impairment rather than sensorimotor deficits, since there is no difference between diabetic mice and normal mice in the performance of swimming speed. Similarly, in the probe test, diabetic mice also showed prominent memory impairment in STZ-induced diabetic mice. These findings corroborate with earlier studies. In addition, we found that FNS administration improved cognitive deficit of diabetic mice, which presented a significant increase in escape latency and obvious increase in percentage in the platform quadrant.

The model of diabetes mellitus is likely to alter motor activity and anxiety-like behaviors which are major parameters of cognitive dysfunction [34]. In order to examine the effect of FNS in cognitive dysfunction in diabetic mice, open field test was carried out [35]. In the locomotor activity of open field test, diabetic mice exhibited locomotor deficit. Besides, the number of standing up times is significantly decreased compared to normal control group. Conversely, increased locomotor activities and standing up times were observed after FNS administration. These results suggested that the FNS reversed diabetes-associated spatial memory impairment as well as increasing locomotion activity and anxiety-like behavior.

The present study analyzed mitochondria change and the synaptic structure as well as the number of the hippocampus in diabetic mice using serial section electron microscopy [24, 36, 37]. We found that both synaptic structure and mitochondria envelope appeared to damage in diabetic mice compared to control mice. Besides, STZ-induced diabetic mice presented significant reductions in synapse number which indicated that the loss of synapses may be caused by cognitive dysfunction. Excitingly, the damage of microstructure was normalized by means of administration of FNS.

The etiology of diabetes and neurological diseases such as Alzheimer's disease (AD) is similar to each other [38]. Among many kinds of sharing mechanisms, oxidative stress is closely linked to the pathogenesis of neurodegenerative impairment and diabetes [39–41]. The generation of free oxygen radicals induced by hyperglycemia could stimulate the production of proinflammatory cytokines and expression of amyloid protein, which may lead to diabetes-associated cognitive deficits [42]. Therefore, measurements of the activity time course of antioxidant enzymes are important for diagnosing diabetes and Alzheimer's diseases. The results of the present study demonstrated that diabetes significantly increased MDA levels and decreased the activities of antioxidants SOD and CAT in both hippocampus and liver. However, our study indicated that FNS intake markedly increased SOD and CAT activities by inhibiting the elevation of MDA levels. These findings demonstrated that antioxidation contributed to the therapeutic and preventive effects of FNS on diabetes-induced cognitive impairments.

HPLC and TCL characterization and quantification of FNS revealed the presence of flavonoid with similar construction to scutellarin, which may be responsible for the antidiabetic and antihyperlipidemic activities of FNS. *α*, *α*-diphenyl-*β*-picrylhydrazyl (DPPH) free radical scavenging method offers the first approach for evaluating the antioxidant potential of a compound, an extract or other biological sources [28]. In order to explore whether the active component is scutellarin, we carried out DPPH free radical scavenging assay. In vitro, we demonstrated that the component scutellarin of FNS has a significant antioxidant activity. Strikingly, it has been demonstrated that flavonoids have various positive effects on complex metabolic pathologies including diabetes, through multifarious modes of actions. And this provided the evidence that flavonoid of N-FNS may be the effective composition.

## 5. Conclusion

In conclusion, this study revealed for the first time that FNS extract had positive effects on STZ-induced memory dysfunction. We have shown that FNS was a worthy therapeutic and complementary medicine in treating neuronal disturbances in diabetes, of which the principal mechanism was improving the antioxidant activity of the diabetic mice. Further studies of the effects of FNS on the efficacy and the molecular mechanism as well as potential therapeutic effects are necessary.

## Figures and Tables

**Table 1:** Effect of FNS on the area under the curve (AUC) for glucose during OGTT.

Group	NC	DM	Metformin	L-FNS	H-FNS
AUC (h xmmol/L)	787.5±97.5	2712±112.2^Δ^	1681.5±136.2^*∗∗*^	1948.5±129.75^*∗∗*^	1462.5±139.2^*∗∗*^

The values are reported as the means ± SD (n = 10). ^Δ^*P*< 0.001 versus NC, ^*∗*^*P*<0.05, ^*∗∗*^*P<*0.01 versus DM.

## Data Availability

The data used to support the findings of this study are included within the article.

## Additional Points

*Highlights.* (i)* Fragaria nilgerrensis *Schlecht. presents good antihyperglycemic effects on streptozotocin- (STZ-) induced mice. (ii)* Fragaria nilgerrensis *Schlecht. and its constituent scutellarin have potential antioxidant effects against reactive oxygen species (ROS). (iii)* Fragaria nilgerrensis *Schlecht. possesses significant neuroprotective effects on STZ-induced mice.
